# Dydrogesterone is an eligible tool to suppress LH surge in assisted reproduction technologies (ART) cycles

**DOI:** 10.5935/1518-0557.20250003

**Published:** 2025

**Authors:** Marcelo Marinho de Souza, Maria do Carmo Borges de Souza, Roberto de Azevedo Antunes, Ana Luíza Barbeitas, Verônica de Almeida Raupp, Ana Luísa Bruno Marinho de Souza, Layna Almeida Barbosa da Silva, Ana Cristina Allemand Mancebo, Flávia Fernandes Sequeira

**Affiliations:** 1 Fertipraxis - Human Reproduction Center. Barra da Tijuca, Rio de Janeiro - RJ, Brazil; 2 Federal University of Rio de Janeiro - Gynecology Departament of the Clementino Fraga Filho Universitary Hospital. Cidade Universitária da Universidade Federal do Rio de Janeiro, Rio de Janeiro - RJ, Brazil

**Keywords:** Dydrogesterone, GnRH agonist, LH surge, metaphase II oocytes, *in vitro* fertilization

## Abstract

**Objective:**

To evaluate Dydrogesterone’s effectiveness in PPOS protocols for IVF/ICSI or oocyte cryopreservation, focusing on LH surge suppression and metaphase II oocyte yield.

**Methods:**

A retrospective, comparative, single-center study of 550 IVF/ICSI and 186 oocyte cryopreservation cycles was conducted from January 2018 to December 2020. Exclusion criteria included endometriosis, previous ovarian surgery, ovarian insufficiency, and abnormal FSH/LH levels. Patients received either Follitropin delta (Rekovelle^®^) or Menotropin (Menopur^®^). LH surge blockade was achieved with GnRH antagonist (Cetrotide^®^) or DYG (Duphaston^®^). Primary outcome was incidence of premature LH surge; secondary outcomes included follicle size on hCG day, metaphase II oocytes, cancelled cycles, and OHSS. ANCOVA analyses were used, with partial squared Eta as the effect size index.

**Results:**

Premature LH peak with early follicular rupture occurred in 2 cases in Group 1 (Ant) and 3 cases in Group 2 (DYG), without statistical significance. LH levels on trigger day showed no difference (Ant: 2.63 - SD 1.15 *vs*. DYG: 2.47 - SD 1.22). Oocyte maturation at metaphase II (MII) stage was similar (6.28 - SD 4.72 *vs*. 6.71 - SD 4.53). Follicle size differed: fewer follicles ≥ 18 mm in Ant group (3.33 - SD 2.06 *vs*. 4.19 - SD 2.53; *p*=0.001), but more 15-17 mm follicles (*p*=0.024). No moderate to severe OHSS occurred in patients with AMH >3 ng/mL.

**Conclusions:**

DYG is an eligible tool for IVF/ICSI cycles intended to freeze-all and oocyte preservation, embryo banking, and preventing OHSS in patients with high AMH levels.

## INTRODUCTION

In recent decades, the landscape of assisted reproductive technologies (ART) has witnessed significant progress, driven by the refinement of embryo and egg vitrification techniques. This progress has prompted the continuous enhancement of various controlled ovarian stimulation (COS) regimens, a critical aspect influencing oocyte recovery and overall outcomes (Hossein Rashidi *et al.*, 2020). Despite these advancements, the persistent challenge of premature luteinizing hormone (LH) peaks in ovarian stimulation protocols remains a focal point in reproductive medicine. Historically, up to 30% of controlled ovarian stimulation cycles were at risk of cancellation due to premature LH peaks leading to untimely ovulation before oocyte retrieval (Hossein Rashidi *et al.*, 2020; Iwami *et al.*, 2018; Kuang *et al.*, 2015).

Additionally, while traditional protocols have effectively addressed premature LH surges in *in vitro* fertilization (IVF)/intracytoplasmic sperm injection (ICSI) and oocyte vitrification treatments, they have fallen short in mitigating the risk of ovarian hyperstimulation syndrome (OHSS) and managing costs effectively. This limitation has sparked continued interest in the exploration of novel treatment regimens (Xiao *et al.*, 2014; Zhu *et al.*, 2017a).

Despite being a well-known approach, the use of a GnRH agonist (GnRH-a) for pituitary gland desensitization poses challenges such as an extended usage period, high doses, and elevated costs. In contrast, GnRH antagonists (GnRH-ant) have demonstrated effectiveness in preventing premature LH peaks, offering enhanced safety against OHSS, and requiring fewer injections per cycle, while maintaining high treatment costs (Hossein Rashidi *et al.*, 2020; Iwami *et al.*, 2018; Kuang *et al.*, 2015; Pirard *et al.*, 2006; Xiao *et al.*, 2014; Zhu *et al.*, 2017a). In response to these challenges, recent years have witnessed the emergence of new regimens aiming for improved efficacy and patient convenience.

Notably, two significant publications in 2016 shed light on the LH-suppressing effects of exogenous progestins (PGs), sparking interest in their potential role as an alternative to GnRH analogues for preventing premature LH peaks (Li *et al.*, 2016; Wang *et al.*, 2016). However, initial studies raised concerns about the negative impact of PGs on endometrial receptivity, making their application impractical. With the evolution of vitrification techniques and improved oocyte and embryo survival rates, the landscape has shifted, bringing attention back to PGs as a promising option, especially in the context of social fertility cryopreservation and ‘freeze-all’ protocols (La Marca & Capuzzo, 2019).

The first randomized trial emphasizing Progestin-Primed Ovarian Stimulation (PPOS) in 2015 evaluated medroxyprogesterone acetate (MPA) due to its mildly androgenic action (Kuang *et al.*, 2015). Subsequent studies explored the role of MPA, comparing it with Ganirelix in oocyte donation cycles, while other types of PGs, such as natural micronized progesterone (NMP), were still investigated for their effectiveness in preventing premature LH peaks (Beguería *et al.*, 2019; Zhu *et al.*, 2015).

Building on the efficacy and safety established by MPA and NMP, researchers delved into the potential benefits of DYG, a retroprogesterone with a selective PG receptor agonist profile, in the context of COS. Comparative studies, including one by Zhu *et al.* (2017b), concluded that DYG effectively blocks premature LH surges, demonstrating similar results in embryonic quality and pregnancy rates compared to other PGs.

This comparative study aims to evaluate the effectiveness of DYG in PPOS protocols for IVF/ICSI cycles and oocyte cryopreservation, assessing its capacity to block premature LH peaks and its impact on the quality of recovered oocytes.

### Physiological principles of using progesterone to block LH surge

While estradiol (E2) traditionally plays a predominant role in the feedback control of gonadotropin secretion during the follicular phase, some studies in contraceptive research suggest the efficacy of progestins (PGs) in blocking the LH surge and preventing ovulation (Massin, 2017; Messinis, 2006). Experiments in monkeys, whose cycle regulation mechanisms are similar to that occurring in women, the administration of a progestin (levonorgestrel) from the beginning of the cycle prevents the occurrence of the LH surge despite the increase in circulating E2 levels for as long as its use is continued, being a completely reversible action with its interruption. The same experiment was conducted after previous destruction of the hypothalamus. Under these conditions, the administration of E2 valerate induced the LH surge within 24 hours, proving to be a pituitary action. In contrast, the addition of PG was unable to block the E2-induced LH elevation, indicating that PG inhibition of this event occurs at the level of the hypothalamus (Wildt *et al.*, 1981).

Similarly, Chabbert-Buffeta *et al.* (2000) described the action of PG in sheep as an important modulator of LH secretion by reducing the frequency of GnRH pulses which, in turn, enriches the gonadotropic cells in FSH and prevents a second peak of LH. This study demonstrates that changes in PG levels produce dramatic neuroendocrine changes in the frequency of GnRH pulses, quickly reversible after normalization of PG (Chabbert-Buffeta *et al.*, 2000).

Such information supports a new aspect in the control of gonadotropin secretion, with the use of PG from the beginning of the follicular growth stimulus. The timing of its onset is important, as some authors investigated whether this hormone participates in cooperating with the onset of the E2-induced LH peak in the middle of the cycle (Hoff *et al.*, 1979). This concept is confirmed by Zhu *et al.* (2015), who demonstrated that the pituitary block is efficient after 6 days of PG use.

## MATERIAL AND METHODS

A retrospective, comparative single-center study was conducted at Fertipraxis - Human Reproduction Center, a private clinic in the city of Rio de Janeiro, Brazil, from January 2018 to December 2020. The study included 550 in vitro fertilization (IVF)/intracytoplasmic sperm injection (ICSI) cycles and 186 oocyte cryopreservation cycles with LH blockade performed with ant-GnRH or DYG, with no age restrictions. The following exclusion criteria were adopted: patients with endometriosis, previous ovarian surgery, and those with ovarian insufficiency and abnormally high FSH/LH levels. Ethical approval for the study was obtained from the Institutional Review Board (project code 76866123.9.0000.5275), and all participating patients provided written informed consent before the study was conducted.

### Ovarian stimulation

Follicular growth stimulation commenced between the 2^nd^ and 5^th^ day of the menstrual cycle. Patients received Follitropin delta (Rekovelle^®^, Ferring Pharmaceuticals) or Menotropin (Menopur^®^, Ferring Pharmaceuticals) with an individualized daily subcutaneous dose, adjusted as needed based on the attending physician›s assessment using ultrasound monitoring of follicular growth. Clinical decision determined the administration of a GnRH-ant (CTA-Cetrorelix acetate, Cetrotide^®^, Merck) at 0.25 mg/day, initiated flexibly when a follicle reached ≥14 mm and continued throughout the stimulation period (326 cycles). Alternatively, dydrogesterone (DYG) at 10 mg every 8 hours (Duphaston^®^, Abbott) was combined with gonadotrophin from the beginning of stimulation until the day after the trigger (410 cycles). Final follicular maturation occurred with three or more follicles ≥17 mm, triggered by either 250 µg recombinant hCG (Choriogonadotropin alfa, Ovidrel^®^, Merck Serono) or GnRH agonist, 2 ampules (triptorelin acetate Gonapeptyl Daily^®^, Ferring Pharmaceuticals). Cancellation criteria included no follicles with a diameter of 17 mm by day 15, and oocyte retrieval took place 35 hours after triggering.

### Oocyte recovery

The procedure was performed with the patient sedated, using a single-gauge 17-needle (Wallace ONS1733), properly adapted to the vaginal transducer. Aspiration was conducted in a closed-circuit system using an aspiration pump (Pioneer Pro-Pump OS 483) under a pressure of 90-100 mmHg.

### Main outcomes

The primary outcome was the incidence of premature LH surge. Secondary outcomes included metaphase II oocytes, follicles ≥15 mm and <18 mm and ≥18 mm on trigger day and ovarian hyperstimulation syndrome (OHSS) symptoms.

### Statistical analysis

Six analyses of covariance (ANCOVAs) were performed to assess statistically significant differences in scores (LH at trigger, MII, MI, or germinal vesicle (GV) oocytes, follicles ≥18 mm, and follicles ≥15 mm and <18 mm) between two groups, after controlling for age, anti-Mullerian hormone (AMH), antral follicle count (AFC), and body mass index (BMI). Partial squared Eta was used as the effect size index.

Resampling procedures (bootstrapping; 1000 resamplings, with 99% confidence interval) were implemented for group comparison analyses to enhance result reliability and present a 99% confidence interval for means and standard deviations (Haukoos & Lewis, 2005). The analyses were conducted using SPSS software for Windows version 23. Data were expressed as mean ± standard deviation (SD), and a *p*-value ≤ 0.05 was considered statistically significant.

## RESULTS

A total of 736 cycles were distributed into two groups: 326 in the GnRH antagonist group (Ant) and 410 in the Dydrogesterone group (Dyd). Demographic and descriptive characteristics are summarized in Table 1.

**Table 1:** Demographic and treatment characteristics.

	Ant group (n 326)	DYG group (n 410)
Age	37.13±3.94	37.81±3.43
Stimulation days	10.03±2.11	10.15±1.82
AMH	1.95±1.95	1.90±2.51
AFC	12.48±7.63	12.29±9.58
15 - 18 mm	6.25±3.75	5.16±4.66
≥ 18 mm	3.33±2.06	4.19±2.53
BMI	25.45±5.56	24.36±4.05
LH at trigger	2.63±1.15	2.47±1.22
MII	6.28±4.72	6.71±4.53
MI	0.62±1.30	0.47±1.07
GV	1.62±2.28	1.40±2.23

Data expressed as averages ± SD.

In 2 cases in group 1 (Ant) and in 3 cases in group 2 (DYG), a premature LH surge with early follicular rupture were observed, which did not represent a statistical difference (*p*=1.00). The serum LH levels of these patients were excluded from the analysis to avoid skewing the mean value and standard deviation. According to Table 2, the analysis of LH levels on the trigger day revealed no statistical difference between the groups (Ant: Average (Ave) = 2.63, Standard Deviation (SD) = 1.15; DYG: Ave = 2.47, SD = 1.22, *p*=0.19). Controlling for control variables, including age, AMH, AFC, and BMI, we identified no interferences in the evaluated outcomes.

**Table 2:** Analysis of difference between LH scores on trigger in the antagonist and dydrogesterone groups.

Group	M (IC)	SD (CI)	gl	F	p	x^2^
Antagonist	2.63 (2.43 - 2.81)	1.15 (1.05 - 1.23)	1, 273	1.48	0.22	0.005
Dyd	2.47 (2.28 - 2.69)	1.22 (2.28 - 2.69)				

*SD* = standard deviation; Ave = average; GL = degree of freedom; *p* = statistical significance; x^2^ = partial squared Eta.

Control variables: age, AMH, AFC and BMI.

In Table 3, after controlling for the mentioned covariates, there was no statistically significant difference in the number of metaphase II (MII) oocytes. The analysis demonstrated that the Antagonist group presented Ave = 6.28 and SD = 4.72 of MII oocytes, while the Dydrogesterone group presented Ave = 6.71 and SD = 4.53, with no significant difference between the groups (*p*=0.15).

**Table 3:** Analysis of difference between MII, MI and GV scores between the antagonist and dydrogesterone groups.

Variable	Group	M (CI)	SD(CI)	gl	F	p	x^2^
M II	Antagonist	6.28 (5.48 -7.14)	4.72 (3.95 - 5.42)	1, 289	2.12	0.15	0.007
	Dydrog	6.71 (6.00 - 7.48)	4.53 (3.87 - 5.11)				
VG	Antagonist	1.62 (1.25 - 2.02)	2..28 (1.75 - 2.72)	1, 250	0.12	0.73	0.001
	Dydrog	1.40 (1.04 - 1.79)	2.23 (1.68 - 2.69)				
MI	Antagonist	0.62 (0.44 - 0.83)	1.30 (0.75 - 1.76)	1, 251	0.25	0.61	0.001
	Dydrog	0.47 (0.31 - 0.66)	1.07 (0.62 - 1.53)				

SD= standard deviation; *Ave* = average; gl = degree of freedom; *p* = statistical significance; η

2 = partial squared Eta.

Control variables: age, AMH, AFC and BMI.

Similarly, concerning the analysis of metaphase I (MI) and germinal vesicle (GV) oocytes, after controlling, no statistically significant differences were observed between the groups (Table 3).

Regarding the number of follicles ≥ 18 mm and 15 to 17 mm, which was not the primary outcome of the study but a possible indication of the quality of stimulation with different protocols, the analysis demonstrated that the Antagonist group showed lower rates of follicles ≥ 18 mm (Ave = 3.33, SD = 2.06) than the Dydrogesterone group (Ave = 4.19, SD = 2.53) - *p*=0.001. Additionally, the Antagonist group exhibited higher rates of 15 to 17 mm follicles (Ave = 6.25, SD = 3.75) compared to the Dydrogesterone group (Ave = 5.16, SD = 4.66) - *p*=0.024 (Table 4).

**Table 4:** Analysis of difference between ≥ 18 mm and 15 to 18 mm follicles scores between antagonist and Dyd groups.

Variable	Group	Ave (CI)	SD (IC)	gl	F	*p*	*x* ^2^
≥ 18 mm	Antagonist	3.33 (3.03 -3.62)	2.06 (1.81 - 2.29)	1, 289	13.50	0.001	0.05
	Dydrog	4.19 (3.71 - 4.66)	2.53 (2.20 - 2.83)				
15 to 17 mm	Antagonist	6.25 (5.67 - 6.90)	3,75 (3.30 - 4.17)	5,151	5.15	0.024	0.02
	Dydrog	5.16 (4.45 - 5.89)	4.66 (4.18 - 5.19)				

*SD* = standard deviation; Ave = Average; GL = degree of freedom; *p* = statistical significance; *x*^2^ = partial squared Eta.

Control variables: age, AMH, AFC and BMI.

## DISCUSSION

The evaluation of progestins (PG) as alternatives to GnRH agonists and antagonists in preventing the luteinizing hormone (LH) surge during ovarian stimulation cycles has gained attention due to ease of use and potential cost savings. Kuang *et al.* (2015) conducted a prospective controlled study assessing the efficacy of medroxyprogesterone acetate (MPA) since the day 3 of the PPOS cycles, comparing this protocol with conventional short a-GnRH protocol. The study showed a longer stimulation by 1 day and a lower total gonadotropin dose in the MPA group, around 400 IU, compared to the a-GnRH protocol. Despite these differences, the number of mature oocytes was similar in both groups of normal responders (Kuang *et al.*, 2015). Wang *et al.* (2016) still evaluating the effects of MPA, this time in patients with polycystic ovary syndrome (PCOS), reported in a randomized controlled study that MPA reduces the incidence of premature LH surge without interfering with pregnancy outcomes in IVF/ICSI cycles. More, the data also indicated a reduction in the risks of OHSS in these patients (Wang *et al.*, 2016). Similarly, our study found no statistical difference in the number of mature oocytes between Dydrogesterone and ant-GnRH groups. Even evaluating another type of progestin, Dydrogesterone, our results also showed no statistical difference in the number of mature oocytes, as well as in the LH peak on the day of the trigger. Despite similar results in stimulus duration, AMH and AFC, after controlling for all variables, more favorable distribution was observed, with a higher number of follicles ≥ 18 mm in favor of the Dydrogesterone group, which may suggest potential benefits in terms of ovarian response.

Patients with polycystic ovary syndrome (PCOS) represent a challenge group due to LH hypersecretion, hyperandrogenism, and increased intrafollicular androgen levels, which can affect oocyte quality, lead to lower fertilization rates, higher abortion rates, and increased risk of ovarian hyperstimulation syndrome (OHSS) (Delvigne *et al.*, 1993; Wang *et al.*, 2016). While GnRH antagonists are known to reduce the incidence of moderate and severe OHSS, our study did not observe cases of moderate or severe OHSS in either group. This is consistent with findings by Pundir et al. (2012), who highlighted the high risks in PCOS patients even with GnRH antagonists. Another downside for women with PCOS is their increased cycle cancellation rates for IVF compared to women without PCOS (Heijnen *et al.*, 2006). Although we have not separately evaluated patients with PCOS, we did not observe cases of moderate or severe OHSS, as well as of cycle cancellation in any of the groups.

Beguería *et al.* (2019) studied the effects of MPA with a focus on the assessment of non-inferiority, compared to ganirelix with regard to the number of mature oocytes (MII) in egg donation programs. In a randomized clinical trial, 173 donors were divided into 2 comparison groups and the results were comparable both in the amount of recovered MII and in the pregnancy rates of these eggs (Beguería *et al.*, 2019).

With the discussed reduction of endometrial receptivity in cycles of controlled ovarian stimulation and the improved results of the vitrification technique in the last decade, new stimulation regimens have emerged. Indeed, transfer of frozen embryos in freeze-all protocols has been followed by encouraging results in pregnancy and birth rates (Devroey *et al.*, 2011; Doody, 2014; Wong *et al.*, 2014). In our experience, we have observed an increasing number of older patients and a consequent increase in the performance of embryonic genetic studies, as well as in the preservation of fertility in younger women. For such cases, a protocol with PG is suitable for blocking the LH peak, with a relevant cost reduction.

Several recent studies highlight the effectiveness of natural micronized progesterone (NMP), both in supporting the luteal phase and blocking the LH surge. Zhu *et al.* (2015) demonstrated an efficient block of pituitary LH levels after 6 days of using Utrogestan^®^, with similar results, with no case of premature LH elevation being observed in COH cycles (Zhu *et al.*, 2015).

Taking into account only the cost-effectiveness of Dydrogesterone, given the different treatment models, recent data from 2019 show that the use of progestin was not cost effective compared to GnRH analogues when compared to fresh embryo transfer cycles. However, in freeze-all cycles, progestins were clearly superior in terms of cost-effectiveness. In the same sense, also in relation to the rates of premature births, newborn weight and malformation, no differences were demonstrated between Dydrogesterone and GnRH agonists (Evans *et al.*, 2019). Our work did not cover obstetric outcomes, but they are in frank agreement with respect to cost-effectiveness.

Motaref *et al.* (2020) compared Dydrogesterone and ant-GnRH and their effects on the quality of oocytes and embryos in women undergoing ICSI, highlighting the qualities of PG, not only in preventing the LH surge but also for its supposed ability to improve the number of retrieved oocytes. Although we identified similar results between the protocols, with regard to costs, it seems to us that the choice of Dydrogesterone is fully justified, whenever the planned treatment allows it. Additionally, this work has demonstrated a higher rate of follicles > 18 mm in the Dydrogesterone group, which can be understood as a positive differential of this protocol.

Considering effectiveness, convenience and tolerability, Griesinger *et al.* (2018) emphasized the use of NMP by vaginal route, as the oral route has low bioavailability and is associated with more frequent systemic adverse events. In a large multi-center randomized study, they compared NMP with Dydrogesterone, reporting favorable results to Dydrogesterone which, although not statistically significant, confirm the importance of the drug in the current context. In a randomized controlled trial (RCT), Hossein Rashidi *et al.* (2020), based on the low cost, efficacy and greater practicality of oral presentation, affirm the tenability of using Dydrogesterone as an alternative to GnRH antagonists for the prevention of LH peaks. Likewise, our results were similar to these and other recent studies that compared Dydrogesterone with GnRH antagonists, either for luteal phase support or for PPOS cycles, highlighting its favorable cost-effectiveness, greater convenience of the oral use, lower androgenic activity, adverse effects and good tolerability when compared to other progestins (Hossein Rashidi *et al.*, 2020; Zhu *et al.*, 2017b).

We recognize its retrospective design as the main weakness, which leads to future prospective studies. However, our data revealed similar results with respect to premature LH peak, LH level, number of oocytes in metaphase II of Dydrogesterone group compared to GnRH antagonists, making this synthetic PG a cost-effective tool in IVF/ICSI cycles for freeze-all / PGT-A and oocyte preservation. It can also be considered in egg/embryo bank programs or as prevention of OHSS in patients with high levels of AMH.
